# Macrophage transfer promotes intestinal mucosal healing by encouraging transit-amplifying cell expansion in mice

**DOI:** 10.3389/fimmu.2025.1555695

**Published:** 2025-07-21

**Authors:** Dong su Kang, Young min Song, Yeon Ji Park, Hyeon Jong Jeong, Jung Joo Hong, Seung Hyeok Seok, Yi Rang Na

**Affiliations:** ^1^ Translational Immunology Lab, Department of Transdisciplinary Medicine, Seoul National University Hospital, Seoul, Republic of Korea; ^2^ Immunology Core Facility, Department of Translational Research Center, Biomedical Research Institute, Seoul National University Hospital, Seoul, Republic of Korea; ^3^ Cancer Research Institute, Seoul National University, Seoul, Republic of Korea; ^4^ Macrophage Lab, Department of Microbiology and Immunology, and Institute of Endemic Disease, Seoul National University College of Medicine, Seoul, Republic of Korea; ^5^ Department of Biomedical Sciences, Seoul National University College of Medicine, Seoul, Republic of Korea; ^6^ National Primate Research Center, Korea Research Institute of Bioscience and Biotechnology, Cheongju, Republic of Korea; ^7^ Department of Medicine, Seoul National University College of Medicine, Seoul, Republic of Korea

**Keywords:** mucosal immunology, regeneration, macrophage, IMC, cell-therapy, DSS-induced Colitis model

## Abstract

Cellular therapy, including stem cell injections, has been proved to be therapeutic for patients with inflammatory bowel disease (IBDs), showing promising outcomes of disease progression. However, challenges of stem cell therapy remain, such as Crohn’s disease with complex fistula, thus limiting its use and requiring another cellular therapy target for efficacy. Alternatively, macrophages have been reported to enhance recovery of damaged intestinal epithelial barriers during resolution of IBDs; thus, utilizing macrophage as a therapeutic strategy was hypothesized. In this study, we compared the regenerative capacity of wild-type and *Tnf^−^* macrophages to validate the potential of genetically modified macrophages with low-inflammatory properties. Our findings demonstrate that *Tnf^−/−^* macrophage transplantation ameliorates weight loss and shortening of colon in a mouse model of colitis. Imaging mass cytometry revealed that *Tnf^−/−^* macrophages particularly increase the population of transit-amplifying cells. Cellular interaction analysis further identified a subset of fibroblast to be in proximity to these epithelial cell types. Collectively, this brief study suggests that phenotype-modified macrophage transplantation facilitates mucosal healing in IBDs, which is supporting evidence for potential cellular therapy in IBD patients.

## Introduction

Inflammatory bowel diseases (IBDs) are chronic intestinal disorders, with most patients diagnosed with either Crohn’s disease (CD) and ulcerative colitis (UC) (1). Incidence reports of IBDs have been increasing, showing a significant increase in their prevalence worldwide, requiring attention as a global burden (2). With medical advancements, therapies for IBDs have been progressing, especially with immunomodulatory therapies such as antitumor necrosis factor alpha (anti-TNFα) (3, 4). While these therapies have made great advancements for treating IBDs, non-responding patients and loss of response to the drug still hold significant proportions of IBD patients, requiring further research for effective treatment.

Cellular therapy employing autologous stem cells for patients afflicted with IBDs is actively undergoing clinical trials (4, 5). However, challenges of stem cell therapy remain in the spectrum of IBDs, such as Crohn’s disease with complex fistula (6). Moreover, the underlying mechanisms of action associated with this therapeutic approach remain uncertain, requiring further investigations for refinement and enhancement of stem cell therapy. In the meantime, macrophages have been gaining attention as promising cellular candidates for cell-based therapies. For instance, autologous macrophage therapy has entered the phase II clinical trial for patients with liver cirrhosis (7), whereas chimeric antigen receptor (CAR)–macrophages have received FDA approval for HER2^+^ breast cancer (8). Notably, macrophages play critical roles in regulating the mucosal environment during IBDs, particularly in modulating inflammation and promoting healing in such pathological conditions (9–11). Recent advancements in research highlight the phenotypic changes of macrophages during inflammation (12–14), and the importance of this innate immune cell in achieving complete remission of IBDs has become increasingly recognized. Macrophages with an anti-inflammatory profile have been identified as a potential therapy target for IBDs, as this phenotype releases pro-resolving factors (15–18). Collectively, macrophages could present a promising cell-based therapeutic strategy for IBDs, but which cells they interact with and how it impacts other regenerative machinery, including epithelial cells and other immune cells, remains unclear (19).

Under IBDs, immunomodulation has been recognized as a critical factor for positive results (3, 4, 20). During stem cell therapy, a combination of anti-inflammatory treatment resulted in an increase of cell therapy efficacy (21), suggesting the importance of immunomodulation of cytokines for effective IBD treatment. Given the versatile nature of macrophage phenotypes, we considered conceivable side effects arising from the additional synthesis of TNF by transferred macrophages. Thus, we initially hypothesized that utilizing genetically modified TNF-deficient (*Tnf^−^* ^/−^) macrophages may be more effective in promoting intestinal regeneration in comparison with unmodified naïve macrophages in condition of IBDs. Furthermore, we hypothesized that therapeutically administered macrophages might stimulate intestinal regeneration by coordinating crucial cell-to-cell interactions. However, there has yet been any spatial evidence that suggests which types of cells primarily interact with regenerating epithelial cells. To confirm these hypotheses, we conducted spatial analysis using highly multiplexed imaging known as imaging mass cytometry (IMC) (22). Here, we report conserved colon length and weight change in IBD mouse models treated with *Tnf^−^* ^/−^ macrophages, and IMC analysis reveals increased transit-amplifying cells closely associated with a specific fibroblast population, indicating mechanistic insight of macrophage cell therapy for IBDs.

## Methods

### Animal information

All animals were purchased from Jackson Laboratory and bred under specific pathogen-free conditions at the animal facility of the Seoul National University College of Medicine. All mice were fed a standard lab chow diet with access to water and food ad libitum. All mice were used 10–12-week-old male B6.SJL-Ptprca Pepcb/BoyJ (CD45.1, Jackson #002014) were used for colitis modeling. C57BL/6J (wild type) and B6.129S-Tnftm1Gkl/J (TNF-α KO, Jackson #005540) were used for bone marrow-derived macrophage extraction, to gather *Tnf^−^* ^/−^ macrophage. For GFP^+^ macrophages, C57BL/6-Tg (CD68-EGFP) 1Drg/J (hCD68-GFP, Jackson #026827) was used for bone marrow-derived macrophage extraction. Animal experiments were conducted in accordance with them and were cared for according to the Guide for the Care and Use of Laboratory Animals prepared by the Institutional Animal Care and Use Committee (IACUC) of Seoul National University (accession number SNU-201214-3-12).

### DSS-induced colitis modeling

A DSS-induced colitis model was made by following protocols from Na et al. (15), with modifications. Except for the control group, all mice subjected for the colitis model were given 3% dextran sulfate sodium (DSS) (MP Biomedicals, California, USA) drinking water (w/v) for 5 consecutive days, replacing fresh DSS-infused drinking water once on the third day. Then, normal water was given during a 2-day recovery period. Mice were weighed each day to determine weight change. On day 7, mice were euthanized by a CO_2_ chamber and intestines were sampled. From the appendix to the rectum, colon lengths of each group were compared and measured. Colons about 1 mm below the appendix was sampled for cytometric bead array assay (<1-mm length obtained), and the rest were harvested and Swiss-rolled for immunohistochemistry or imaging mass cytometry. For each treatment group (control, DSS, DSS + WT BMDM, DSS + *Tnf^−^* ^/−^BMDMs) we used N= 5–6 mice.

### Bone marrow harvest and macrophage differentiation

Bone marrow (BM) cells were obtained from wild-type (C57BL6), hCD68-GFP, or TNF-a KO mice. All mice were euthanized using the CO_2_ chamber, and hindleg femurs were collected removing muscles and skin with surgical tools. Femur tips were cut open, flushed with plain RPMI medium, and kept at 4°C into a 50-mL conical tube. Then, tubes were centrifuged at 1,300 rpm for 5 min. Supernatant was removed, and cells were resuspended in 6 mL of ammonium–chloride–potassium (ACK) lysing buffer (4 M potassium bicarbonate, 0.5 M ammonium chloride in 1× PBS), prewarmed in a 37°C water bath for 30 min, and incubated up to 5 min for red blood cell removal. The ACK buffer was stopped by adding 1× PBS up to 50 mL and then were centrifuged at 1,300 rpm for 5 min. Cells were resuspended into 1 mL culture medium (10% FBS, 1% P/S, in RPMI) and were counted. 5 × 10^6^ cells were seeded into a T-75 flask, in culture medium added with macrophage colony stimulating factor (M-CSF) at 100 ng/mL for 5 days for BM-derived macrophages (BMDMs), following protocols from Na et al. (15). Some of the cell cultures were maintained to be used for *in vitro* quantitative PCR experiments. For harvest of bone marrow, N= 4 were used for each WT and *Tnf^−^* ^/−^ mice. For GFP BMDM, N=3 mice were used.

### Adoptive macrophage cell transfer

BMDMs differentiated from three strains of mice were harvested from a cell culture flask using trypsin and resuspended in cold PBS. 1.5 × 10^6^ cells of either WT or *Tnf^−^* ^/−^BMDMs were adoptively transferred into CD45.1 mice by intraperitoneal (i.p) injection on the 3rd day of DSS treatment, for designated experimental groups only. For GFP^+^ BMDMs, cells were transferred at 2 × 10^6^ via I.P injection.

### Histological staining: immunohistochemistry and H&E

To validate adoptive macrophage transfer, GFP^+^ BMDM-injected mouse intestines were harvested and fixed overnight in 4% paraformaldehyde (PFA). For positive control, hCD68-GFP mouse intestines were also obtained. After fixation, tissues were moved to 30% sucrose with 0.01% sodium azide solution and incubated at least 24 h or more for cryopreservation. Tissues were embedded in an OCT compound and were frozen using dry ice, and a cryosection of 8- to 10-μm slide was obtained. Slides were permeabilized with 0.3% Triton X-100 and blocked using 3% BSA, 0.3% Triton-X in 1× PBS for 30 min. The slides were incubated in F4/80 (Abcam, 1:400) in antibody solution (1% BSA, 0.1% Triton-X) in 4°C overnight. Then, the slides were washed and incubated in Goat anti-Rat IgG (H+L), Alexa Fluor™ 555 (1:2,000, Invitrogen, A-21434), and DAPI (1:1,000, Invitrogen, 62248). Slides were mounted with VECTASHIELD anti-fade mounting medium and imaged using a confocal microscope (*LEICA, SP5*), and a z-stack of 10-μm image was obtained. Images were analyzed using JaCOP Plugin from ImageJ to determine co-localization quantification using Mander’s coefficient for quantification of GFP^+^F4/80^+^ cells.

For H&E staining, a 5-μm slide was obtained from the cryosection and was immediately washed with 1× PBS three times to remove the OCT compound. Then, hematoxylin was applied for 3 min and washed in running distilled water, and then eosin was applied for 1 min. Afterward, slides were dipped for 1 min into 95% ethanol and 100% ethanol twice, no longer than 1 min each for the dehydration process. Afterward, slides were further dehydrated in xylene for 1 min twice and were dried and mounted with a coverslip using Permount Mounting Medium (Fisher Chemical, SP15-100). The slide image was taken using a brightfield microscope (ECLIPSE Ci-L, Nikon).

The histological score on the H&E slide image was performed based on the following grading system, which is the same grading system used from Na et al. (15): inflammation, 0 = no changes; 1= infiltrations in the lamina propria; 2 = extending into the submucosa; 3 = transmural extension; epithelial damage, 0 = no change; 1 = loss of the basal one-third; 2 = loss of the basal two-thirds; 3 = entire crypt loss, 4 = epithelial erosion, 5 = confluent erosion; mucosal architecture, 0 = no changes; 1 = 1 or 2 foci of ulcerations, 2 = 3 or 4 foci of ulcerations, 3 = confluent or extensive ulceration. For each treatment group (control, DSS, DSS + WT BMDM, DSS+ *Tnf^−^* ^/−^BMDMs) we used N= 5 mice.

### *In vitro* experimentation

To determine how macrophages may influence fibroblast change, fibroblast cell line NIH3T3 was thawed and cultured for 7 days under DMEM with 10% FBS and 1% P/S (cDMEM). Then, NIH3T3 cells were seeded with a density of 5 × 10^4^ in a 24-well plate, creating triplicates for each of the following groups: control, WT macrophage supernatant treated, or *Tnf^−^* ^/−^ macrophage supernatant treated. After 24 h, mouse BMDMs differentiated before the experiment had media replaced with fresh 10% FBS, 1% P/S, in RPMI containing M-CSF, and supernatant was collected 24 h later. For the experiment, we first made a mixture of media containing 1:1 of either cDMEM and WT macrophage supernatant or *Tnf^−^* ^/−^ macrophage supernatant. Each conditioned medium was given to each well of triplicated cultures accordingly. Then, either 6 or 12 h later, cells were washed with PBS, and RNA was harvested using a TRIzol and chloroform mixture.

### cDNA synthesis and quantitative PCR

For measurement of mRNA expression from fibroblasts, we first synthesized cDNA from the RNA extracted from above. We used ReverTra Ace qPCR RT Master Mix (Toyobo, FSQ-201) to perform cDNA synthesis as instructed within the product. Then, using SYBR Green (Enzynomics, RT500M), we used primers for gene expression: *Arg1* (forward: 5′CTGGAACCCAGAGAGAGCAT3′, reverse: 5′CTCCTCGAGGCTGTCCTTT3′), *Col1a1* (forward: 5′GAGAGCATGACCGATGGATT3′, reverse: 5′CCTTCTTGAGGTTGCCAGTC3′), and *Mmp9* (forward: 5′CCCTACTGCTGGTCCTTCTGAG3′, reverse: 5′AATTGGCTTCCTCCGTGATTCG3′), with *Rps18* (forward: 5′GCAATTATTCCCCATGAACG3′, reverse: 5′GGCCTCACTAAACCATCCAA3′) as a reference gene. We used QuantStudio 6 to run the quantitative PCR and used obtained values to analyze and normalize the relative mRNA expression level.

### Slide preparation for imaging mass cytometry

Mouse intestines used for imaging mass cytometry were preserved in 30% sucrose in 1× PBS for 2 h for cryopreservation, embedded in an OCT compound (Sakura, Cat# 4583), and flash frozen in liquid nitrogen. The cryosection was performed, and 5-μm section slides were obtained. Slides were kept at −80°C until the staining procedure. Prior to staining, slides were stabilized at −20°C for 1 h.

### Antibody conjugation and imaging mass cytometry

For IMC, 12 antibodies were conjugated using the Maxpar X8 Multimetal Labeling Kit (Fluidigm) and others were pre-conjugated antibodies (**Supplementary Table 1**). A total of 31 antibody panels were used for staining. All steps performed by following staining protocol were available on Fluidigm (FLDM-00073 Rev 01 PROTOCOL). All Maxpar^®^ X8 Antibody Labeling Kits for antibody metal conjugation kits were purchased from Standard BioTools as follows: 144Nd (Cat# 201144A), 150Nd (Cat# 201150A), 155Gd (Cat# 201155A), 156Gd (Cat# 201156A), 158Gd (Cat# 201158A), 161Dy (Cat# 201161A), 163Dy (Cat# 201163A), 171Yb (Cat#201171A), 172Yb (Cat#201172A), 173Yb (Cat#201173A), 174Yb (Cat#201174A), 175Lu (Cat#201175A). Image acquisition was performed using the Hyperion Tissue Imager (Fluidigm) and CyTOF software (Fluidigm, version 7.0.8).

For the staining process, IMC slides were first fixed in 4% PFA at 4°C for 30 min prior to any staining procedures. Slides were washed in Maxpar PBS for 10 min and then blocked with 3% BSA in Maxpar PBS and 0.3% Triton-X for 30 min at room temperature. While blocking, antibody cocktail containing all antibodies listed in **Supplementary Table** at 1:300 dilution was prepared using 0.5% BSA and 0.3% Triton-X in Maxpar PBS solution. The antibody was centrifuged at 13,000*g* for 2 min and pipetted before use to avoid antibody aggregates. After removing blocking solution, slides were incubated in antibody cocktail at 4°C overnight. Then, slides were washed with 0.1% Tween 20 in 1× PBS for 8 min twice. Then, tissues were incubated with Intercalator-Ir (1:1,000) in PBS for 1 h at room temperature. Finally, the slides were washed in Maxpar water for 5 min and air dried for at least 20 min. The slides were kept in a slide box until acquisition. Image acquisition was performed using the Hyperion Tissue Imager (Fluidigm) and CyTOF software (Fluidigm, version 7.0.8), with ROI designated to include all anatomical layers of mouse intestine.

### Imaging mass cytometry data analysis

Image data were analyzed following analysis workflow from *Windhager* et al. (23). Raw IMC data were obtained and using Steinbock software, and segmentation of single cells within tissues and single-cell data extraction were performed. Extracted data were converted into SingleCellExperiment objects using the imcRtools and cytomapper packages for downstream analysis.

For comparison of marker expression levels, we applied *asinh* transformation to the raw count data for normalization of marker expressions at a single-cell level. The *Cytomapper* package was also utilized to generate image masks, allowing visualization of spatial distribution of markers within each cell.

For clustering of cell population, we implemented the FlowSOM algorithm as our primary clustering method. Using the *scater* package, we performed dimensional reduction to generate UMAP and t-SNE for better visualizations cell population clusters. The *dittoSeq* package was used to generate heatmaps, which provided a comprehensive visualization of how key markers were expressed across different cell population clusters, identifying distinct cell populations for each cluster. Cytomapper was used again to visually validate clustered populations within tissue.

For neighborhood analysis to determine intercellular relationships, we constructed spatial interaction graphs through a combination of k-nearest neighbors (KNN) analysis, Delaunay triangulation, and distance-based expansion methods. Then, using the “testInteractions” function, we evaluated spatial relationships between different cell types identified. These data were normalized and visualized to reveal the complex cellular organization within the tissue microenvironment.

### Statistical analysis

Prism 9.5.1 was used for statistical analysis. All data that were quantified and compared were analyzed statistically using either one-way or two-way ANOVA or t-test by using GraphPad Prism. While parametric tests were generally used, the non-parametric Kruskal–Wallis test was applied for selected graphs where appropriate, with details provided in the respective figure legends. Data in all graphs are presented as the mean + standard error of the mean (SEM). All statistical tests held a <0.05 for significance.

## Results

### TNF-deficient macrophage transfer mitigates pathology of a DSS-induced IBD mouse model

**Figure 1:**
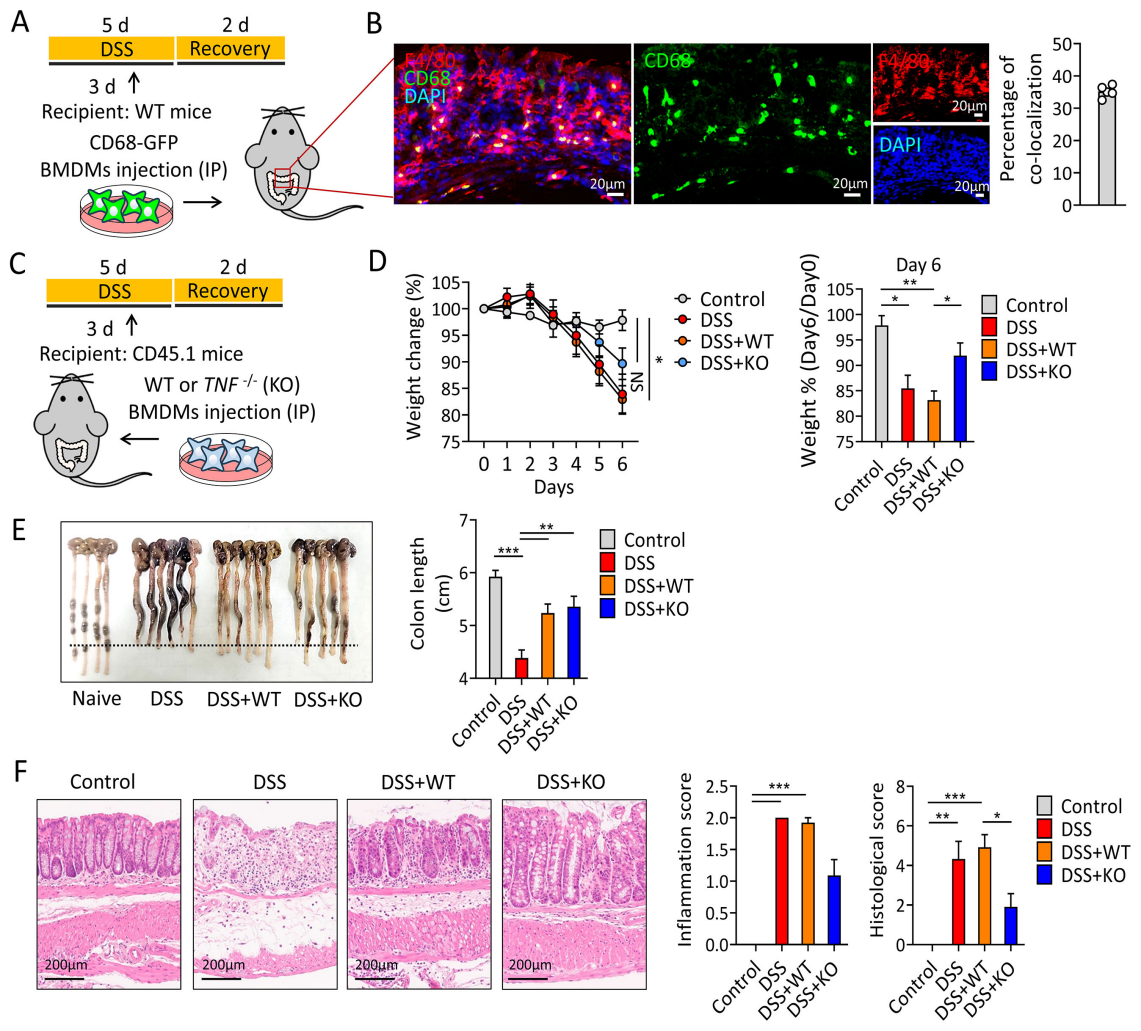
Macrophage transfer improves outcomes in the DSS colitis mouse model. **(A)** Schematic of the DSS colitis model and macrophage transfer. Mice were given 3% of DSS in drinking water for 5 days. BMDMs from CD68-GFP mice (5 × 10^6^ cells) were intraperitoneally injected on day 3 of DSS supply. **(B)** Representative IF image showing CD68 (green), F4/80 (red), and nuclei (blue). *Scale bar =20 μm.* **(C)** Schematic of WT and *TNF^−^* ^/−^ BMDM transfer. **(D)** Left, body weight changes over 7 days. DSS treatment began on day 0, after baseline weight measurement. Right, graph showing relative weight proportion on day 6 of DSS supply. **(E)** Comparison of colon lengths and corresponding quantification. Colons were harvested on day 7 post-DSS treatment, and lengths were quantified. **(F)** Left, H&E staining of colon sections from each group. *Scale bar = 200 μm*. Middle, graph showing the inflammation score. Right, graph showing the histological score. Kruskal–Wallis test with Dunn’s *post hoc* test was used. DSS, dextran sodium sulfate; BMDMs, bone marrow-derived macrophages; WT, wild type, IF, immunofluorescence, and H&E, hematoxylin and eosin. N= 5–6 per group. One-way ANOVA, *p<0.05, **p<0.01, ***p<0.001.

To validate regenerative effects of macrophage transfer, we first verified the *in-situ* migration and persistence of the injected macrophages in inflamed intestine (**Figure 1A**). Bone marrow-derived macrophages (BMDMs) from hCD68-GFP mice were intraperitoneally (i.p) injected on day 3 of IBD mouse modeling. GFP-expressing macrophages were readily observed in the mucosal layer of colon (**Figure 1B**), predominantly colocalized with F4/80 expression, which was densely distributed near the crypt.

Next, ex-vivo differentiated CD45.2^+^ wild-type (WT) and *Tnf^−^* ^/−^ macrophages were injected (i.p) into CD45.1 host mice (**Figure 1C**). CD45.2^+^ macrophages were detected in the mucosal layer of the host mouse colon (**Supplementary Figure S1**). On day 6 post-DSS treatment, when weight loss in the IBD mouse model reached its peak (15), the *Tnf^−^* ^/−^ macrophage group (*KO*), but not the WT macrophage group (*WT*), showed reduced weight loss compared with its IBD control group (DSS only, *IBD ctrl*) during the colitis phase, suggesting a protective effect against colitis-induced weight loss (**Figure 1D**). Interestingly, both *KO* and *WT* significantly preserved the colon length, suggesting their pro-regenerative effects compared with the *IBD ctrl* (**Figure 1E**). Histological analysis revealed that mice from *KO* could conserve the mucosal layer of the colon from inflammatory responses (**Figure 1F**), whereas *WT* could not. Taken together, ex-vivo generated naïve macrophages could migrate to inflamed areas and promote tissue regeneration, although their impact on the inflammatory status is insignificant, whereas *Tnf^−^* ^/−^ macrophages not only promote tissue regeneration but also reduce inflammation.

### Imaging mass cytometry reveals an increase in transit-amplifying cells following the injection of TNF-deficient macrophages

**Figure 2:**
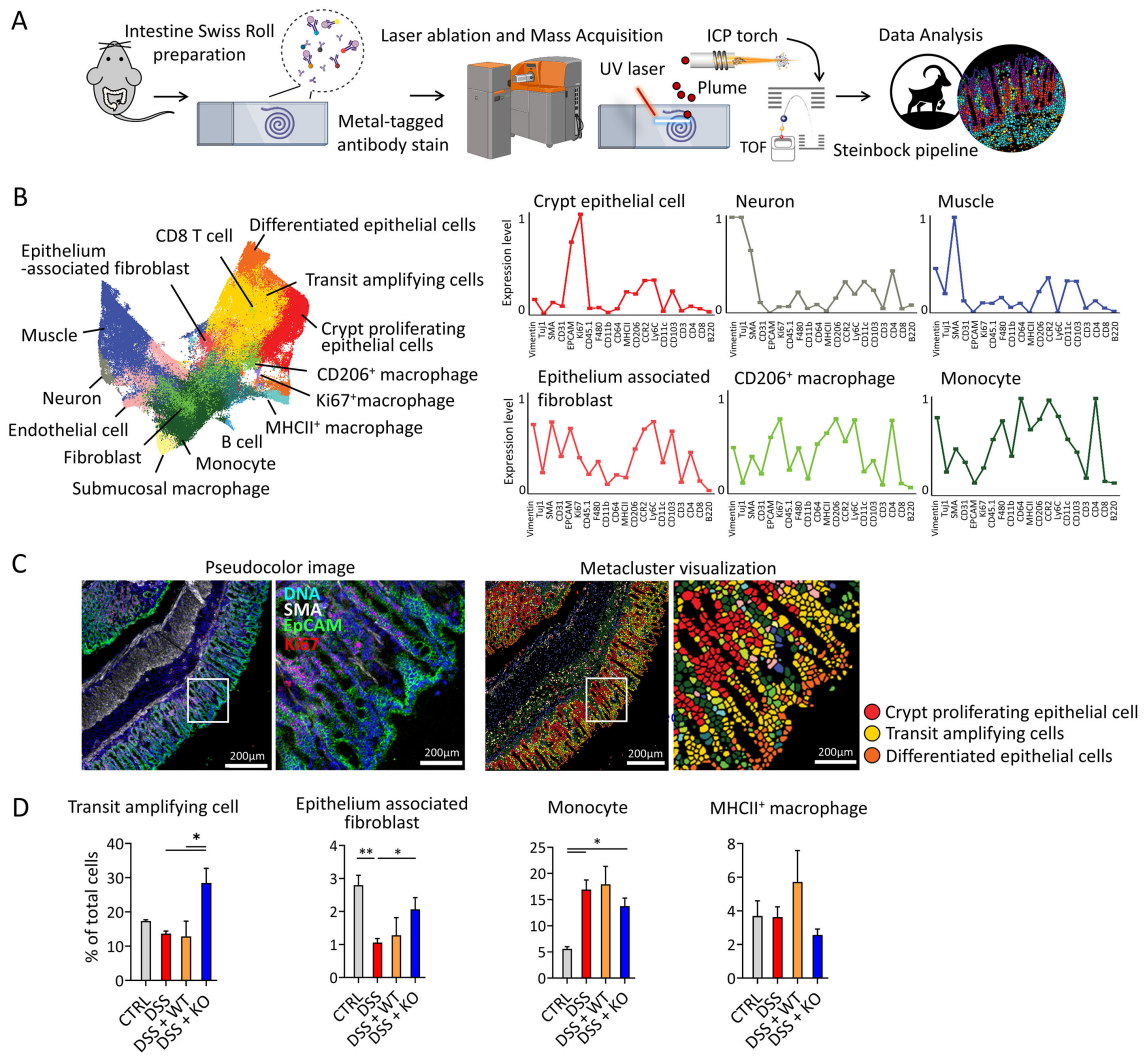
Imaging mass cytometry reveals different cell subtypes and compositions in colons of mice with adoptive macrophage transfer during colitis. **(A)** Overview of IMC process. The colon was formed into a Swiss roll and prepared by cryotome cross section, with 5-µm thickness on the glass slide. IMC, imaging mass cytometry. *N= 5–6 per group*. **(B)** UMAP plot of mouse colon of all four groups. Unsupervised single-cell clustering was performed using FlowSOM. A total of 15 identified clusters were labeled on the right. Each cluster is divided into cell type identified by marker expression on the heatmap (**Supplementary Figure S3**). Right line graph representing the expression level within cell type identified by IMC. **(C)** Image of identified subtypes of different epithelial cells based on marker expressions. Pseudocolored image (left) and single-cell segmented image (right) are matched; *Scale bar = 200 µm*. Images were zoomed in on an area (white square) to visibly observe different types of epithelial cells based on marker expressions (*right of each image; scale bar = 50 µm*). **(D)** different cell types were compared between each group for total cell percentage, which had significant differences. MHCII^+^ macrophage is shown to represent pro-inflammatory activity of macrophages. One-way analysis of variance (ANOVA) *p<0.05; **p<0.01 N=5–6 per group, three ROI per mice.

After confirming regenerative effects from macrophage transfer, we investigated spatial changes in the mouse colons using high-plex imaging mass cytometry (IMC) with a 32-metal conjugated antibody panel (**Figure 2A**; **Supplementary Table**). For IMC, we selected two regions of interest (ROI) per mouse, with each ROI (1 mm^2^) encompassing all layers of intestine, from mucosa to muscular layers. Structural markers including EPCAM, SMA, vimentin, and CD31 readily delineated histological layers, whereas immune cell markers distinguished macrophages, monocytes, T cells, and B cells within mucosa-associated lymphoid tissue (**Supplementary Figure S2**). Next, we investigated cellular composition within ROIs through single-cell segmentation and FlowSOM clustering. Each ROI identified on average of 8,050 single cells, and based on expression levels of 32 different markers, each cell was assigned into one of 15 distinct clusters (**Figure 2B**). CD45.2 was not included, as clustering was performed on all ROIs collected from four experimental groups, of which two did not receive adoptive macrophage transfers. Characterization of each cluster identified epithelial cells, immune cells, neuronal cells, muscle cells, fibroblasts, and endothelial cells (**Supplementary Figure S3**). Further subcharacterization identified three discrete epithelial cell types based on EPCAM and Ki67 expression: crypt proliferating epithelial cell (Ki67^hi^EPCAM^+^), transit-amplifying cell (Ki67^int^EPCAM^+^), and differentiated epithelial cells (Ki67^lo^EPCAM^hi^) (**Figure 2C**). In addition, macrophages were identified into four different subtypes, based on expressions of Ly6C, CD206, MHCII, CX3CR1, CD64, CD11b, CD11c, and F4/80 as follows: MHCII^+^ macrophage, CD206^+^ macrophage, submucosal macrophage, and ki67^+^ macrophage (**Supplementary Figure S3**). Fibroblasts are marked by high SMA expression and divided into two clusters, EPCAM^high^ epithelium-associated fibroblast and EPCAM^low^ fibroblast, suggesting their location (**Figure 2B**; **Supplementary Figure S3**).

After categorizing all cellular subtypes, we determined population proportions of each cell type in different experimental groups (**Supplementary Figure S4**). Our results showed that under normal conditions, Ki67^lo^EPCAM^hi^-differentiated epithelial cells are dominant among all epithelial cell types, composing 20% of total cells. Meanwhile, immune cells make up 14% of total cells, of which 40% were monocytes. Within the DSS group (*IBD ctrl*), dominant epithelial cell type changed, with differentiated epithelial cell decreasing by half and Ki67^hi^EPCAM^+^ crypt proliferating epithelial cells becoming relatively dominant, making up 18% of total cells. Meanwhile, the immune cell population increased to approximately 30% on average, and the CCR2^hi^ monocyte population showed a noticeable expansion, reaching up to threefold in some cases. When examining the effects of macrophage injection, mice injected with *TNF* knockout (KO) macrophages exhibited a significant increase in transit-amplifying epithelial cells (**Figure 2D**), suggesting a role of *TNF-*deficient macrophages in promoting epithelial renewal (24, 25). Inflammation was associated with a reduction in epithelium-associated fibroblasts relative to naive mice; however, this population was significantly restored in the KO macrophage-injected group compared with the DSS group. Additionally, the KO group showed a trend toward lower proportions of monocytes and MHCII^+^ macrophages compared with the WT macrophage-injected group, although these differences were not statistically significant (**Figure 2D**). Overall, our results suggest that *Tnf^−^* ^/−^ macrophage transfer facilitates epithelial regeneration.

### Epithelium-associated fibroblasts establish regional components of actively regenerating epithelium

**Figure 3:**
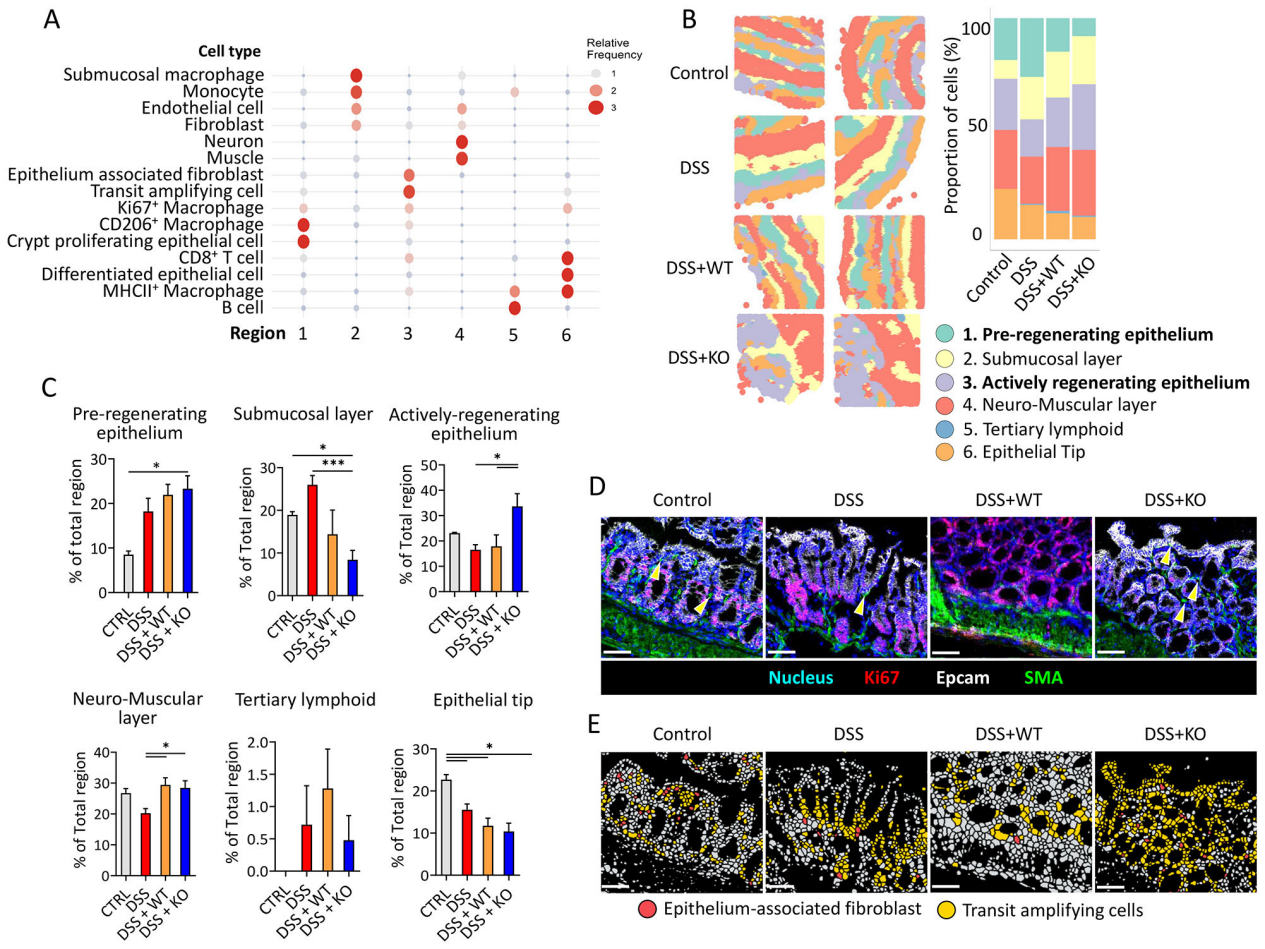
Regional analysis reveals *Tnf^−^* ^/−^ macrophage treatment has increased actively regenerating regions. **(A)** Region annotation showing enriched cell types within six distinct regions. Higher relative frequency indicates enrichment of such cells within each region. **(B)** Spatial imaging of cellular neighborhood analysis based on six identified regions. All images corresponded to the ROI image obtained from IMC; the bar graph on the right shows the proportion of each region per group *N= 5–6 per group.* **(C)** Graph showing the proportion of total regions in **(B)** based on each ROI across all groups. t-test, *p<0.05; ***p<0.01. **(D)** Representative image obtained from IMC of epithelium-associated fibroblast (yellow arrow) and transit-amplifying cells. *Scale bar = 50 µm* **(E)** Representative image of single cell-segmented imaging data from **(D)** highlighting epithelium-associated fibroblast (red) and transit-amplifying cells (yellow) cluster. *Scale bar = 50 µm N=5–6 per group, three ROI per mice*.

As we have identified distinct epithelial cell types and immune cells change post *Tnf^−^* ^/−^ macrophage treatment, we further investigated to understand changes within intercellular associations. We performed cellular neighborhood analysis to determine spatial associations of different cell types; particularly, we focused on which cells are spatially associated with transit amplifying cells as it marked regenerating epithelial barriers during IBD. Through usage of local indicators of spatial associations (LISA) functions (26), which allows identification of distinct regions of tissue that are enriched for combinations of identified cell types, we categorized six regions, with each region representing different microenvironments (**Figure 3A**). Within these, we determined two regions (regions 1 and 3) associated with regenerating tissue area, one muscular layer (region 4), one epithelial tip (region 6), and two inflammation-associated tissue areas (regions 2 and 5). These regions were mapped onto processed ROI image (**Figure 3B**) and were analyzed to determine how macrophage treatment changed regional proportions. Intriguingly, we discovered that *KO* had significantly high proportions of region 3 (**Figure 3C**). This region, mostly enriched with epithelium-associated fibroblasts and transit-amplifying cells, were identified as an “actively regenerating” region. Meanwhile, the KO group also showed a statistically significant increase in the proportion of region 1 compared with the naïve control, whereas the IBD control and WT groups exhibited a similar trend without reaching statistical significance. Region 1 was enriched with CD206^+^ macrophages and crypt epithelial cells and was identified as a “pre-regenerative” region. Both types of macrophage treatment had minimal loss of neuromuscular layer regions compared with *IBD* ctrl. Interestingly, no changes were found in the tertiary lymphoid region in all colitis models (**Figure 3C**), which suggest that macrophage transplant has minimal impact in its formation.

We next confirmed our neighborhood analysis visually by spatial imaging, especially regarding proximity of cells associated with actively regenerating region within IMC images. Several studies have reported the crucial role of fibroblast in epithelial homeostasis (27). Thus, we focused on spatial approximation of EPCAM^+^ fibroblast, as it was the most enriched cells in actively regenerating the epithelial region. The results show that epithelium-associated fibroblast, marked by SMA expression, was in proximity with transit-amplifying cells, and this was unanimous within all groups (**Figure 3D**). In the mucosal region, KO exhibited the highest frequency of epithelium-associated fibroblasts, along with a significant enrichment of transit-amplifying cells (**Figure 3E**). These findings support the accuracy of LISA-clustered mapping for identifying actively regenerating epithelium, signifying that the transplantation of *Tnf^−^* ^/−^ macrophages enhances the differentiation of epithelial cells, facilitated by the presence of fibroblasts.

### Transit-amplifying cells engage in spatial interactions with fibroblasts and CD8^+^ T cells

**Figure 4:**
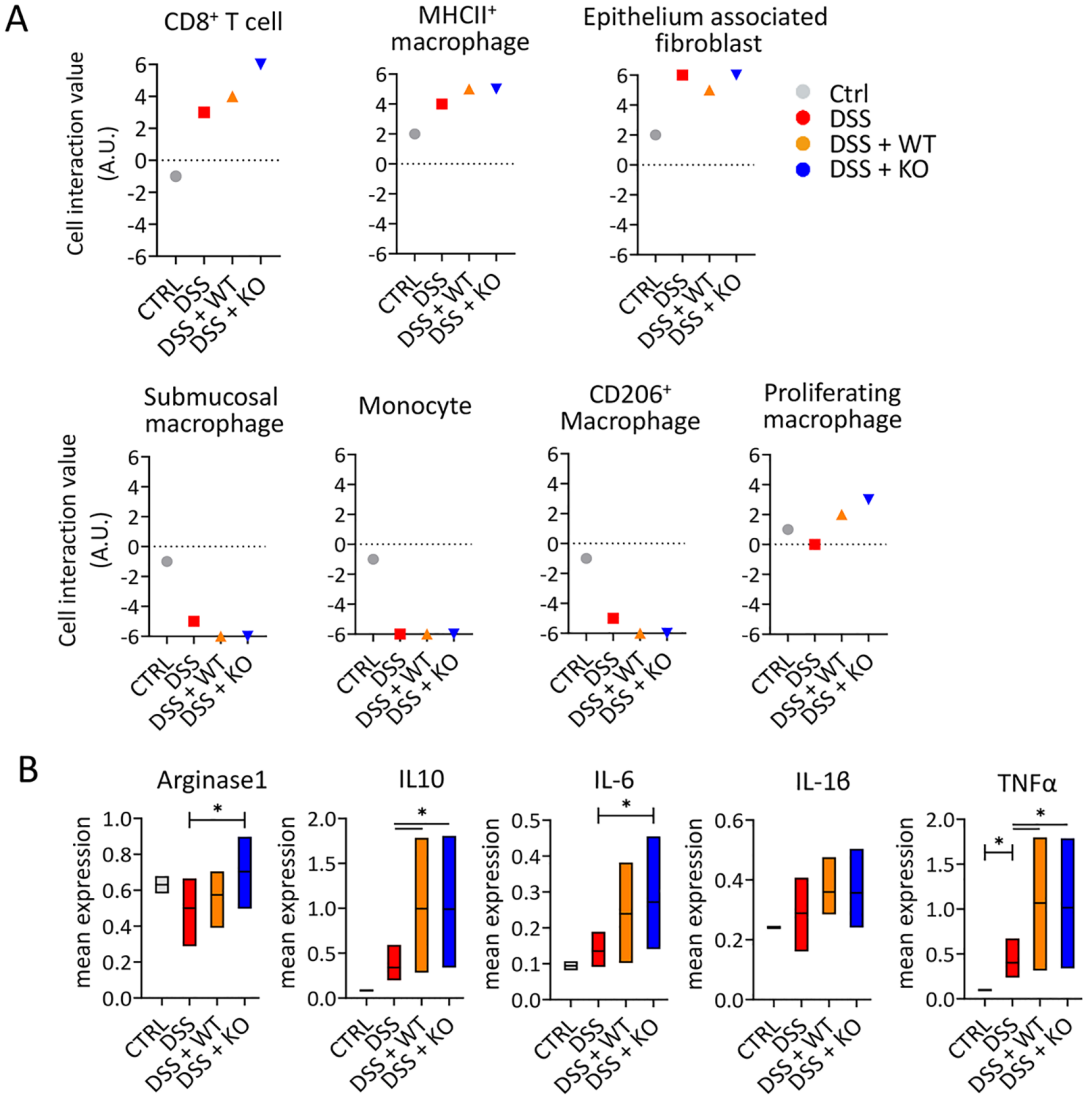
Neighborhood analysis confirms that differentiating epithelial cells are spatially associated with immature macrophages. **(A)** Graph showing the interaction index representing spatial proximity between transit-amplifying cells and various immune cells. Data points are color- and shape-coded as follows: gray circles for CTRL, red squares for DSS, orange upward triangles for DSS+WT, and blue downward triangles for DSS+KO. **(B)** Median cytokine expression plot of five different cytokines by epithelium-associated fibroblast across all groups, obtained from each ROI processed using IMC. t-test, *p<0.05.

To validate whether epithelium-associated fibroblasts were the main cell to interact with transit-amplifying cells, we performed cell-to-cell interaction analysis and focused on proximity of immune cells and fibroblasts. As we selectively analyzed the nearest cell-to-cell proximity against transit-amplifying cells, epithelium-associated fibroblasts were the closest compared with other types of cells in three DSS groups (**Figure 4A**). Intriguingly, CD8^+^ T cells were within proximity to transit-amplifying cells, especially within *KO*, and did not show this pattern in the non-pathological control group. When ordering the hierarchy of proximity with transit-amplifying cells among subtypes in other cell types, MHCII^+^ macrophage exhibited the highest proximity. Conversely, monocyte and CD206^+^ macrophages were shown to be distant with transit-amplifying cells.

Given that epithelium-associated fibroblasts were the nearest cell with transit-amplifying cells, we further investigated its phenotype, based on cytokine expression detected through IMC (28). We determined the expression levels of pro-inflammatory cytokines including TNFα, IL-1ß, and IL-6, along with anti-inflammatory cytokines IL-10 and arginase 1 (**Figure 4B**) across all groups. Results showed that under normal conditions, mucosal fibroblast had negligible cytokine expressions except for Arginase 1, which is known for their role in intestinal homeostasis (29). Notably, *KO* mice had a significant increase in Arginase 1 expression in mucosal fibroblasts compared with other colitis model groups, showing the possible interaction between transplanted macrophages and fibroblasts. Across all groups with colitis, mucosal fibroblast had increased pro-inflammatory signals regardless of macrophage transfer, although variations were high within individual mice. Interestingly, both *WT* and *KO* groups showed significantly elevated levels of TNFα and IL-10, indicating that these cells respond to environmental inflammatory signals in a bipolar manner, with IL-10 acting as a key anti-inflammatory cytokine involved in intestinal homeostasis (30, 31).

## Discussion

In this research report, we have gathered evidence that transfer of genetically modified *Tnf^−^* ^/−^ macrophage has a potential therapeutic effect for IBD, through modulation of spatial architecture of regenerative intestinal epithelium. Systemic treatment with *Tnf^−^* ^/−^ macrophages enhanced the interaction between transit-amplifying cells, mucosal fibroblasts, and CD8^+^ T cells, suggesting a supportive role of these macrophages in promoting tissue regeneration and immune modulation. In the intestine, fibroblasts represent a highly heterogenous population of cell types that act as key players in a multitude of processes essential to intestinal function during development, homeostasis, and disease (27). EPCAM^+^ fibroblasts are spatially localized at the crypt top area of the colon, near the crypt apex, alongside transit-amplifying cells. In addition to the well-known crypt fibroblasts, which contribute to the stem cell niche by producing WNT ligands and Rspo3, crypt top fibroblasts (CTF) also play important roles in maintaining epithelial integrity (32–34). Here, we have further defined the spatial relevance of mucosal fibroblasts to epithelial cells, emphasizing their role during the resolution phase of IBDs. Notably, our findings suggest that macrophages, particularly those with a low-inflammatory signature, support the regenerative function of these fibroblasts, contributing to the restoration of tissue homeostasis. In fact, fibroblasts and macrophages are found in close association in several tissues in the steady state, as found by Germain and his colleagues using *in vivo* imaging (35). Macrophages provide PDGFs for the recruitment and survival of fibroblast (36), and reciprocal interaction also existed (37). Our study is limited in investigating exacting roles of injected macrophages on the interaction between transit-amplifying cells and fibroblasts; however, based on our findings, we cannot eliminate the possibilities that transfer of *Tnf^−/−^* macrophage may have modulated phenotypic changes of fibroblasts for acceleration of epithelial regeneration, possibly by Arginase I production (38). In addition, further investigations *in vitro* showed increased MMP9 expression by fibroblasts treated with WT macrophage supernatant (**Supplementary Figure S5**), which is relevant to increased inflammatory colitis within the intestine (39). As cytokines such as TNFa are a key source in directly inducing MMP-9 expression to degrade the extracellular matrix and induce barrier dysfunction, *Tnf^−/−^* macrophage may have been safer as it does not produce TNFa, which may have contributed to epithelial preservation or regeneration. Our revelations provide foundations for future studies to investigate the mechanisms behind how macrophage transplant affect the microenvironment of regenerating epithelial layers of the IBD mouse model.

Beyond our observation on the spatial interaction of mucosal fibroblasts, we identified natural distributions of macrophage subtypes in intestinal tissue. CD206^+^ macrophages are primarily located near crypts, whereas MHCII^+^ macrophages are predominantly found in region 6 (**Figure 3A**), corresponding to the epithelial tip in this study. The distinct distributions of these subsets suggest specific functional roles, potentially acquired during monocyte waterfall (40), depending on their locations. CD206^+^ macrophages have been reported to promote mesenchymal niche cell proliferation via Wnt6 secretion (41) and differentiate from CCR2^+^ monocytes under the influence of fibroblasts near the crypts (42). In contrast, MHCII^+^ macrophages may uptake luminal antigens in a tolerant state, contributing to oral tolerance (43, 44). Another site-specific subtype we identified is the submucosal macrophage population, characterized by high expressions of F4/80 and CD11b but low expression of CX3CR1 (**Figure 3A**; **Supplementary Figure S3**). These macrophages are localized in region 2, closely associated with endothelial cells, fibroblasts, and monocytes. Due to the absence of CX3CR1 expression, we hypothesize that they may originate from infiltrating monocytes, although further studies are needed to better understand their characteristics. Adding markers such as CD4, Timd4 (45), or CD169 (46) to the staining panel could provide additional insights into these cells.

Our study has revealed that CD8^+^ T cells are one of components of region 6, where they function as intraepithelial T cells, playing a critical role in immune surveillance and maintaining the integrity of the intestinal barrier (47). CD8^+^ T cells are well known for their involvement in the pathogenesis of IBD (48, 49), but recent advancements in single-cell analysis suggest that subsets of theses T cells may have distinct functions (50, 51). The unusual finding in the *KO*, where there is a high interaction between CD8^+^ T cells and transit-amplifying cells (**Figure 4A**), likely reflects a natural process where these T cells integrate into the epithelial barrier during the epithelial regeneration phase. However, it remains unclear whether the immobilization of CD8^+^ T cells is a direct effect of transferred macrophages.

Overall, while this study supports macrophage as a therapeutic source for IBD, it also serves as evidence of concept for utilizing genetic modification of innate immune cells for optimal therapeutic efficacy. Using these cells as a therapy may be applied in many clinical settings besides IBD, including liver cirrhosis, alveolar proteinosis, heart failure, and neuronal degenerative diseases (52). Given that such chimerical antigen receptor–macrophage has been making its way into clinical trials (53), there is a growing prospect for the emergence of genetically modified macrophages as a form of cell therapy. This study not only highlights the importance of macrophages in tissue repair but also supports advancing development in modified macrophages to enhance tissue regeneration in IBD.

### Limitations of this study

Although we have demonstrated positive impacts of macrophage transfer into experimental DSS-induced colitis models, our IBD models are limited in representing IBDs that represent autoimmune IBDs such as Crohn’s disease. In addition, we were limited in the transfer method of macrophages; we performed intraperitoneal injections, instead of intramucosal injection, which is a clinical method used in IBD patients. This may have limited the number of transferred macrophages migrating into the mucosal layer, along with systemic delivery into other organs. Also, neutrophil and stem cell (Ly6G and LGR5) detection was excluded due to antibody validation issue. Finally, we did not perform extensive analysis in determining mechanisms behind cell interactions, as our main goal was to obtain single-cell spatial information of immune cells impacting regeneration of epithelial cells. Studies on immune response toward macrophage transfer are necessary to understand the true potential of macrophage transfer for IBD therapy.

## Data Availability

The original contributions presented in the study are included in the article/**Supplementary Material**. Further inquiries can be directed to the corresponding authors.
