# Supplementary material for: Macrophage transfer promotes intestinal mucosal healing by encouraging transit-amplifying cell expansion in mice
Source: Front Immunol. 2025 Jul 21;16:1555695. doi: 10.3389/fimmu.2025.1555695 (PMC12318747; doi:10.3389/fimmu.2025.1555695)
Supplement: Supplementary file 1 [file DataSheet1.docx]

**Supplemental Figures & Annotations:**

| *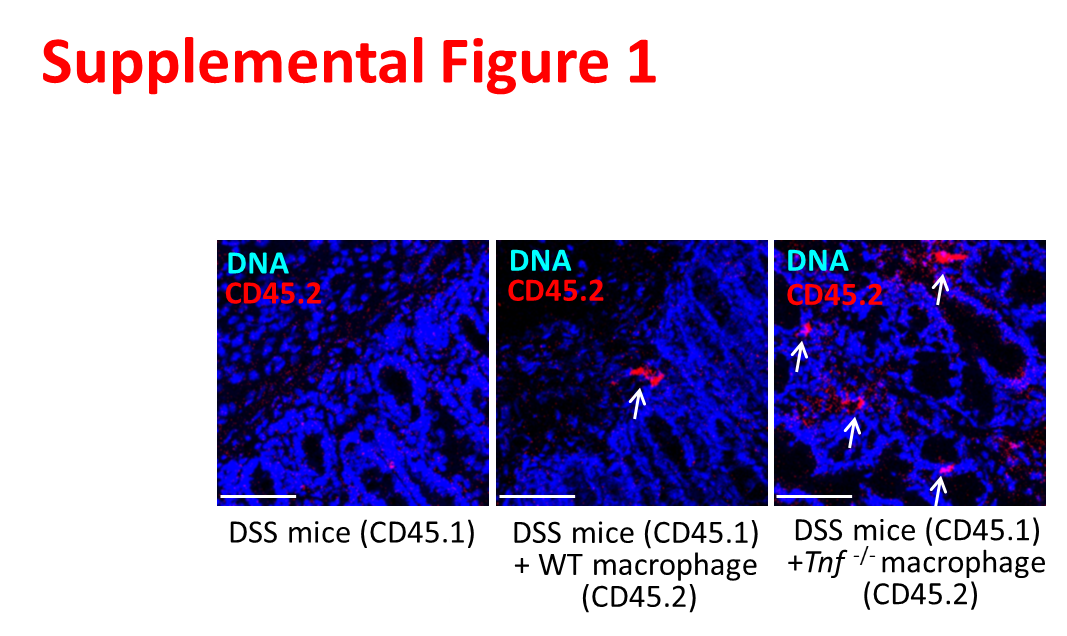* |
| --- |

**Supplementary Figure 1. Transferred macrophages localize to the mucosal layer of colon.** Representative IF image showing CD45.1 (red) and nuclei (blue). CD45.1 mice were given 3% of DSS in drinking water for 5 days. BMDMs from CD45.2 WT and *TNF*^-/-^ macrophages (5X10^6^ cells ) were intraperitonealy injected on Day 3 of DSS supply. Mice were sacrificed on Day 7. White arrows indicate migrated CD45.2^+^ injected macrophages. *Scale bar=100μm.* IF=Immunofluorescence, BMDMs,= bone marow derived macrophages, WT=wildtype. *N=5-6 per group, 3 ROI per mice.*

| *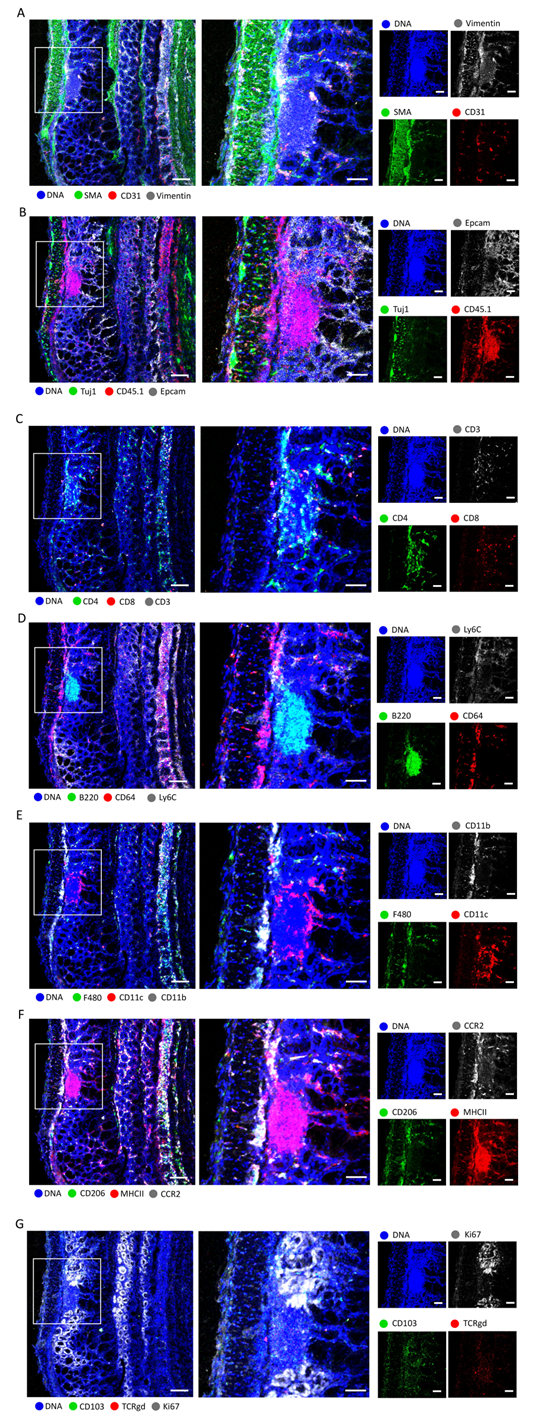* |
| --- |

**Supplementary Figure 2. IMC imaging of tissue biomarker expression in the colon of mouse DSS colitis model. Representative image of IMC data image (1mm^2)^. The image shown here is a colon of DSS + WT macrophage injection.** (A) Left: IMC image of a Swiss role section of colon from DSS-treated mouse with Ir nuclear stain (blue), SMA (green), CD31 (red), and vimentin (gray) shown as a 4-color composite image selected from a total of 32 protein and 2 nuclear markers stained on the same tissue section. Middle: Zoomed-in view of the region denoted in the white box in left image shown as a 4-color composite image. Right: Each of the indicated markers individually (small panels). Scale bar=100 μm. (B) Left: IMC image of a Swiss role section of colon from DSS-treated mouse with Ir nuclear stain (blue), Tuj1 (green), CD45.1 (red), and EPCAM (gray) shown as a 4-color composite image selected from a total of 32 protein and 2 nuclear markers stained on the same tissue section. Middle: Zoomed-in view of the region denoted in the white box in left image shown as a 4-color composite image. Right: Each of the indicated markers individually (small panels). Scale bar=100 μm. (C) Left: IMC image of a Swiss role section of colon from DSS-treated mouse with Ir nuclear stain (blue), CD4 (green), CD8 (red), and CD3 (gray) shown as a 4-color composite image selected from a total of 32 protein and 2 nuclear markers stained on the same tissue section. Middle: Zoomed-in view of the region denoted in the white box in left image shown as a 4-color composite image. Right: Each of the indicated markers individually (small panels). (D) Left: IMC image of a Swiss role section of colon from DSS-treated mouse with Ir nuclear stain (blue), B220 (green), CD64 (red), and Ly6C (gray) shown as a 4-color composite image selected from a total of 32 protein and 2 nuclear markers stained on the same tissue section. Middle: Zoomed-in view of the region denoted in the white box in left image shown as a 4-color composite image. Right: Each of the indicated markers individually (small panels). (E) Left: IMC image of a Swiss role section of colon from DSS-treated mouse with Ir nuclear stain (blue), F480 (green), CD11c (red), and CD11b (gray) shown as a 4-color composite image selected from a total of 32 protein and 2 nuclear markers stained on the same tissue section. Middle: Zoomed-in view of the region denoted in the white box in left image shown as a 4-color composite image. Right: Each of the indicated markers individually (small panels). (F) Left: IMC image of a Swiss role section of colon from DSS-treated mouse with Ir nuclear stain (blue), CD206 (green), MHCII (red), and CCR2 (gray) shown as a 4-color composite image selected from a total of 32 protein and 2 nuclear markers stained on the same tissue section. Middle: Zoomed-in view of the region denoted in the white box in left image shown as a 4-color composite image. Right: Each of the indicated markers individually (small panels). (G) Left: IMC image of a Swiss role section of colon from DSS-treated mouse with Ir nuclear stain (blue), CD103 (green), TCRgd (red), and Ki67 (gray) shown as a 4-color composite image selected from a total of 32 protein and 2 nuclear markers stained on the same tissue section. Middle: Zoomed-in view of the region denoted in the white box in left image shown as a 4-color composite image. Right: Each of the indicated markers individually (small panels). *All images have* *Scale bar=100 μm*. *N=5-6 per group, 3 ROI per mice.*

| 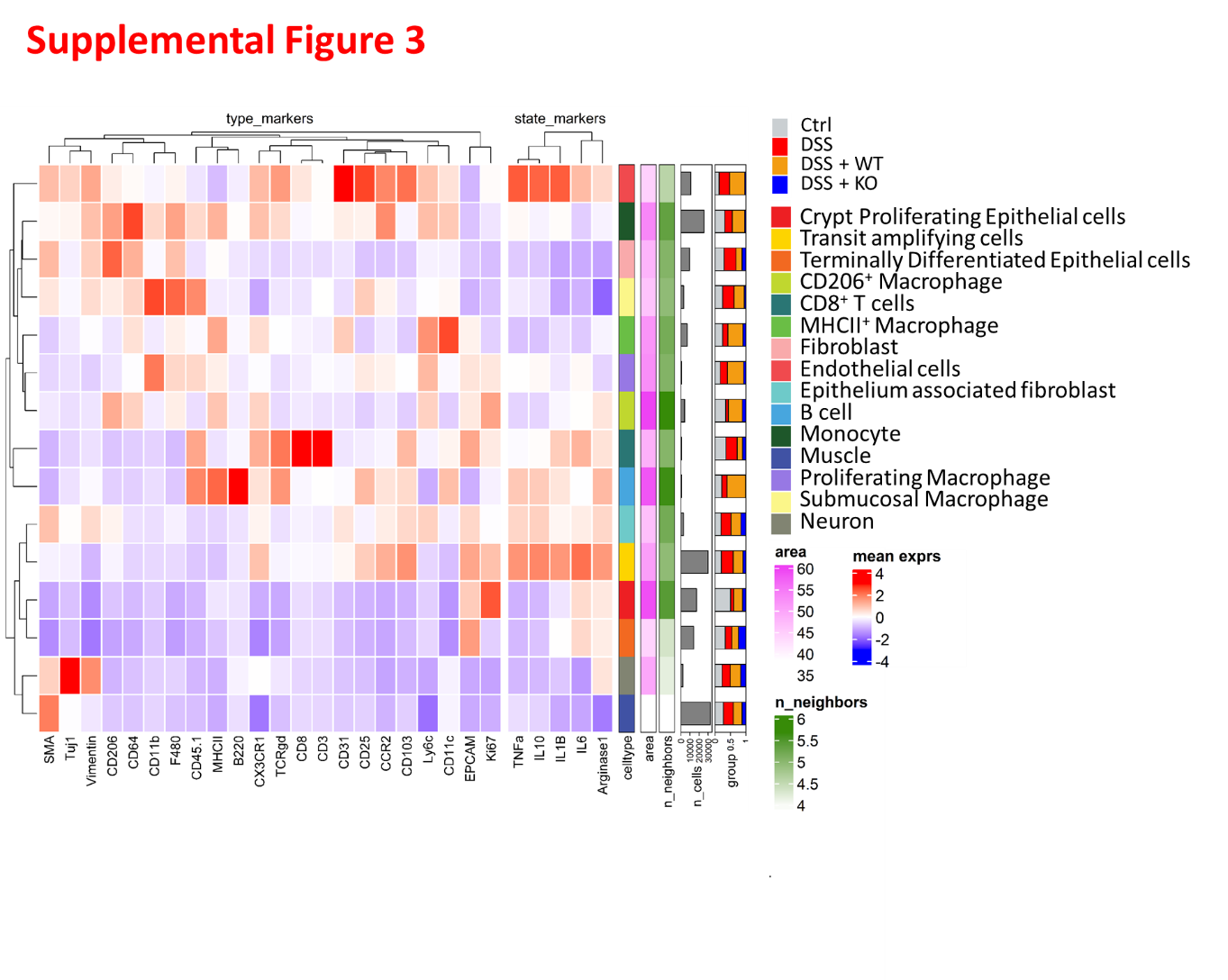 |
| --- |

**Supplementary Figure 3. Identified clusters from IMC imaging based on marker expression reveal 15 distinct cell types.**

Heatmap plot of mouse colon of all four groups. Unsupervised single-cell clustering was performed using Flowsom. 15 identified clusters were labeled on the right. Heat map is divided into cell-type markers and cytokine markers. Right histograms indicate cell type, area present within slide image, number of neighboring cell types (n_neighbors), cell count (n_cells), and abundance proportion in four experimental groups respectively.

|  |
| --- |

**
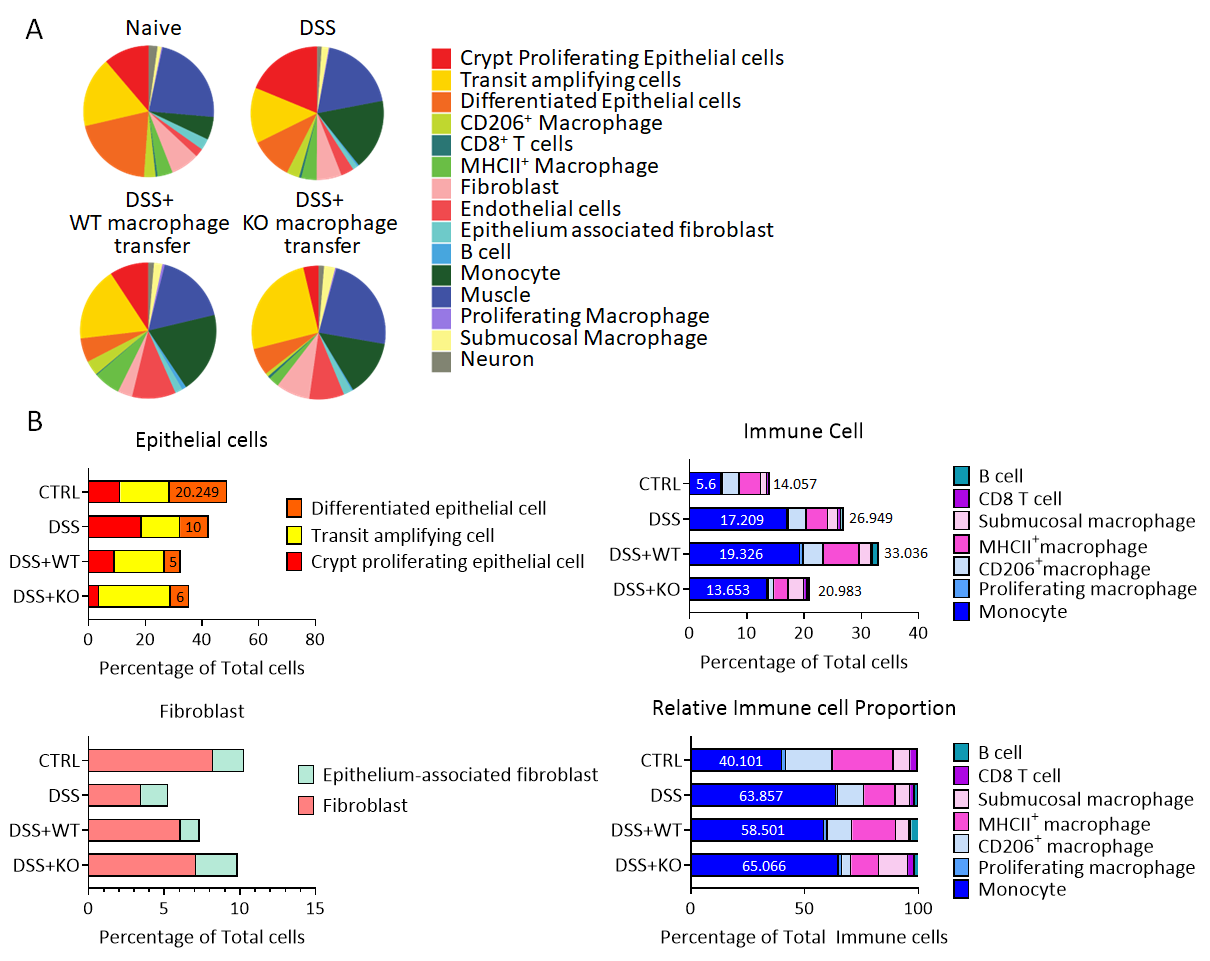
**

**Supplementary Figure 4. Image analysis shows population proportions of individual cell types.** (A) Pie chart showing population proportions of 15 distinct cell types in four experimental groups. (B) Bar graphs illustrating the total proportions of three epithelial subtypes, seven immune cell subtypes, and two fibroblast subtypes. Additionally, relative proportions of immune cell subsets within the total CD45^+^ population are presented. For clarity, absolute percentages corresponding to each bar are indicated within the graphs. All proportions are made based on the percentage of total cells within each experimental group. *N=5-6 per group, 3 ROI per mice.*


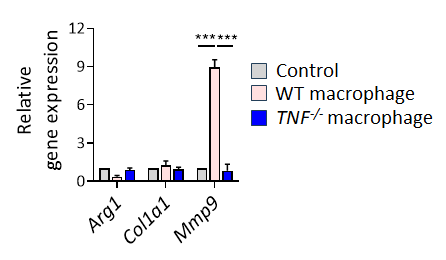


**Supplementary Figure 5. Quantitative PCR analysis of fibroblasts treated with culture supernatants from WT or *Tnf⁻/⁻* bone marrow-derived macrophages.**NIH3T3 fibroblasts were cultured with macrophage-conditioned media for 6 hours (*Arg1, Col1a1*) or 12 hours (*Mmp9*), followed by qPCR analysis. Data represent mean ± SEM. *Two-way ANOVA, ***p<0.001.*

**Supplementary Table 1.**

Antibody panel information based on categorization of set markers used for Imaging Mass Cytometry.

| **Set type** | **Antibody** | **Metal Label** | **Clone** | **Manufacturer** | **Dilution** | **Description** |
| --- | --- | --- | --- | --- | --- | --- |
| **Structure markers** | Vimentin | 143Nd | D21H3 | S.BioTools | 1:400 | Mesenchymal cell cytoskeletal filaments |
|  | SMA | 141Pr | 1A4 | S.BioTools | 1:400 | Muscle cell, Myofibroblast |
|  | CD31 | 165Ho | 390 | S.BioTools | 1:400 | Endothelial cell  (Vascular marker) |
|  | EpCAM (CD326) | 166Er | G8.8 | S.BioTools | 1:400 | Epithelial cell marker |
|  | TUj1 | 144Nd | GT11710 | Genetex | 1:400 | Neuronal cell marker |
|  |  |  |  |  |  |  |
| **Immune cell markers** | CD45.1 | 153Eu | A20 | S.BioTools | 1:400 | Leukocyte marker, Host native |
|  | CD45.2 | 147Sm | 104 | S.BioTools | 1:400 | Leukocyte marker, Injected |
|  | CD11b | 154Sm | M1/70 | S.BioTools | 1:400 | Myeloid lineage marker |
|  | Ly6C | 162Dy | HK1.4 | S.BioTools | 1:400 | Monocyte marker |
|  | CD11c | 142Nd | N418 | S.BioTools | 1:400 | DC lineage marker |
|  | CD3 | 152Sm | 145-2C11 | S.BioTools | 1:400 | T-cell lineage marker |
|  | B220 (CD45R) | 160Gd | RA3-6B2 | S.BioTools | 1:400 | B cell lineage marker (mice) |
|  | F4/80 | 146Nd | BM8 | S.BioTools | 1:400 | Pan Macrophage marker |
|  | CD64 | 151Eu | X45-5 /7.1 | S.BioTools | 1:400 | Macrophage lineage marker |
|  | CD206 | 147Sm | 104 | S.BioTools | 1:400 | Leukocyte marker, Injected |
|  | MHCII | 154Sm | M1/70 | S.BioTools | 1:400 | Myeloid lineage marker |
|  | CD4 | 145Nd | RM4-5 | S.BioTools | 1:400 | CD4^+^ T-cells (Th cells) |
|  | CD8 | 163Dy | 53-6.7 | BioLegend | 1:400 | CD8^+^ T-cells  (Cytotoxic  T- cells) |
|  | TCRgd | 159Tb | GL3 | S.BioTools | 1:400 | Gamma Delta T-Cell marker |
|  | CD103 | 175Lu | 2.00E+07 | Invitrogen | 1:400 | Conventional DC (cDC1) type1 |
|  | CCR2 | 171Yb | EPR20844-15 | Abcam | 1:400 | Recruited Monocyte marker |
|  |  |  |  |  |  |  |
| **Secretion Markers** | IL-6 | 167Er | MP5-20F3 | S.BioTools | 1:400 | Inflammatory Cytokine |
|  | TNFa | 172Yb | MP6-T22 | Invitrogen | 1:400 | Inflammatory Cytokine |
|  | IL-1b | 161Dy | Polyclonal | Invitrogen | 1:400 | Inflammatory Cytokine |
|  | IL-10 | 173Yb | GT5111 | Genetex | 1:400 | Anti-inflammatory Cytokine |
|  | Arginase1 | 156Gd | Polyclonal | NovusBio | 1:400 | Anti-inflammatory Cytokine |
|  |  |  |  |  |  |  |
| **Nuclear Markers** | Ki67 | 168Er | B56 | S.BioTools | 1:400 | Cellular Proliferation |
|  | DNA | 191/193Ir | Intercalator | S.BioTools | 1:400 | Cell nucleus Maker |
