# Supplementary material for: Macrophage transfer promotes intestinal mucosal healing by encouraging transit-amplifying cell expansion in mice
Source: Front Immunol. 2025 Jul 21;16:1555695. doi: 10.3389/fimmu.2025.1555695 (PMC12318747; doi:10.3389/fimmu.2025.1555695)
Supplement: Supplementary file 2 [file Table1.docx]

**Supplementary Table.**

| **ANTIBODIES** | **SOURCE** | **IDENTIFIER** |
| --- | --- | --- |
| Rabbit polyclonal anti-Arginase 1 | Novus bio | Cat# NBP1-32731 |
| Anti-Mouse B220 | Standard BioTools | Cat# 3160012B |
| Rabbit monoclonal anti-CCR2 | Abcam | Cat# ab273061 |
| Hamster monoclonal anti-CD103 | Invitrogen | Cat# 14-1031-82 |
| Anti-Mouse CD11b | Standard BioTools | Cat# 3154006B |
| Anti-Mouse CD11c | Standard BioTools | Cat# 3209005B |
| Anti-Mouse CD206 /MMR | Standard BioTools | Cat# 3165013B |
| Rat monoclonal anti-CD25 | BioLegend | Cat# 101902 |
| Anti-Mouse CD3 (e) | Standard BioTools | Cat# 3152004B |
| Anti-Mouse CD31 | Standard BioTools | Cat# 3165013B |
| Anti-Mouse CD4 | Standard BioTools | Cat# 3145002B |
| Anti-Mouse CD45.1 | Standard BioTools | Cat# 3153002B |
| Anti-Mouse CD45.2 | Standard BioTools | Cat# 3147004B |
| Anti-Mouse CD64 | Standard BioTools | Cat# 3151012B |
| Rat monoclonal anti-CD8 | BioLegend | Cat# 100702 |
| Anti-Mouse CX3CR1 | Standard BioTools | Cat# 3164023B |
| Anti-Mouse EpCAM (CD326) | Standard BioTools | Cat# 3166014B |
| Anti-Mouse F4/80 | Standard BioTools | Cat# 3146008B |
| Rat monoclonal anti-F4/80 | Abcam | Cat# ab6640 |
| Mouse monoclonal anti-IL-10 | GeneTex | Cat# GTX632359 |
| Rabbit polyclonal anti-IL1B | Invitrogen | Cat# P420B |
| Anti-Mouse IL-6 | Standard BioTools | Cat# 3167003B |
| Anti-Mouse Ki67 | Standard BioTools | Cat# 3168022D |
| Anti-Mouse Ly6C | Standard BioTools | Cat# 3162014B |
| Rat monoclonal anti-Ly6G | BioLegend | Cat# 127637 |
| Rat monoclonal anti-MHCII | BioLegend | Cat# 107637 |
| Anti-Mouse SMA (fibroblast) | Standard BioTools | Cat# 3141017D |
| Anti-Mouse TCRgd | Standard BioTools | Cat# 3159012B |
| Rat monoclonal anti-TNFa | Invitrogen | Cat# 3159012B |
| Mouse monoclonal anti-Tuj1 | GeneTex | Cat# GTX631836 |
| Anti-Mouse Vimentin | Standard BioTools | Cat# 3154014A |
| Nucleus marker (Intercalator Ir) | Standard BioTools | Cat# 201192B |

**Supplementary Table.** IMC panels for mouse intestine imaging. All antibodies were used in concentration dilution of 1:300. Nucleus marker (Ir) was diluted in 1:1000. Further information on panels may be available through direct contact, Prof. Yirang Na ([yirangna@snu.ac.kr](mailto:yirangna@snu.ac.kr)).
